# 588. Seroconversion Among Adults After Receiving At Least One Dose of a COVID-19 Vaccine: COVID-19 Community Research Partnership, Mid-Atlantic, Southeast and Southern United States, December 2020-May 2021

**DOI:** 10.1093/ofid/ofab466.786

**Published:** 2021-12-04

**Authors:** DeAnna J Friedman-Klabanoff, Ashley Tjaden, Michele Santacatterina, Iqra Munawar, John W Sanders, David M Herrington, Thomas F Wierzba, Andrea Berry

**Affiliations:** 1 University of Maryland School of Medicine, Baltimore, Maryland; 2 George Washington University, Rockville, Maryland; 3 Wake Forest School of Medicine, Winston-Salem, North Carolina; 4 Wake Forest University School of Medicine, Winston Salem, North Carolina

## Abstract

**Background:**

Well-regulated clinical trials have shown authorized COVID-19 vaccines to be immunogenic and highly efficacious. Information about antibody responses after vaccination in real-world settings is needed.

**Methods:**

We evaluated seroconversion rates in adults reporting ≥ 1 dose of an authorized COVID-19 vaccine in a U.S. multistate longitudinal cohort study, the COVID-19 Community Research Partnership. Participants were recruited through 12 participating healthcare systems and community outreach. Participants had periodic home-based serologic testing using either a SARS-CoV-2 nucleocapsid and spike IgM/IgG lateral flow assay (63% of participants) or a SARS-CoV-2 spike IgG enzyme-linked immunosorbent assay (37% of participants). The timing and number of tests before and after vaccination varied based on participant time in study. Participants were included if they were seronegative on the last test before and had >1 test result after vaccination (some had previously been seropositive, but seroreverted). A weighted Cox regression model with right censoring was used to obtain adjusted hazard ratios for sex, age, race/ethnicity, and prior seropositivity. Time-to-event (seroconversion) was defined as time to first positive test > 4 days after vaccination; participants were censored at the date of their last available test result.

**Results:**

13,459 participants were included and 11,722 seroconverted (Table). Median time in study was 272 days (range 31–395). Median follow-up time from vaccine to last available test was 56 days (range 1–147). Participants had a median of 3 tests (range 1–12) before and 2 tests (range 1–8) after vaccination. Based on the Kaplan-Meier method, median time to seroconversion after first COVID-19 vaccination was 35 days (interquartile range: 25–45). Likelihood of seroconversion decreased with older age (Table). Female participants, non-Hispanic Black participants, and participants who were previously seropositive were more likely to seroconvert (Table).

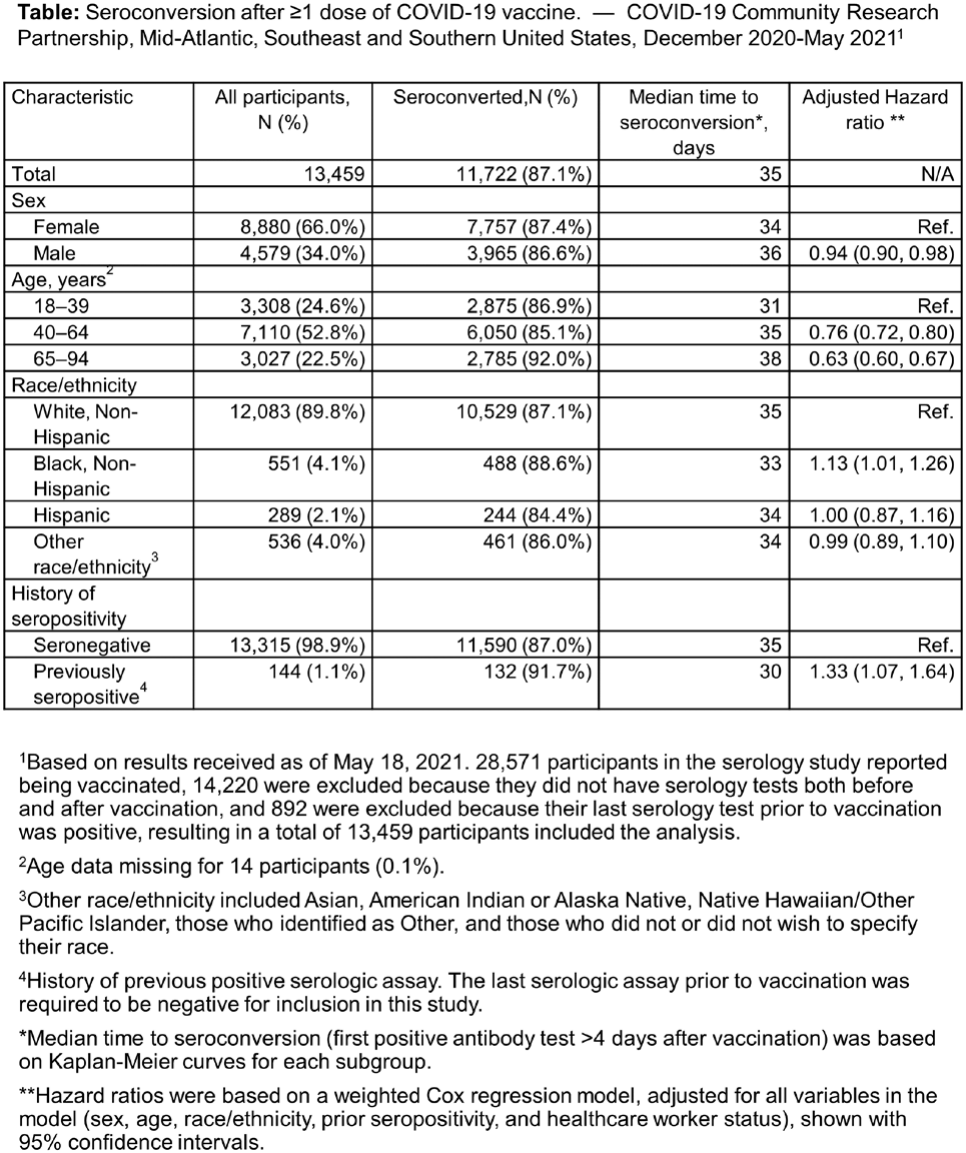

**Conclusion:**

All subgroups had high rates of seroconversion, with some small differences in likelihood of seroconversion between subgroups. These data demonstrate the excellent immunogenicity of COVID-19 vaccines in real-world settings in the US.

**Disclosures:**

**All Authors**: No reported disclosures

